# Types and influencing factors of psychological health among freshmen in Chinese art vocational colleges: a latent profile analysis

**DOI:** 10.3389/fpsyg.2026.1831906

**Published:** 2026-06-17

**Authors:** Shaoyue Chen, Qian Xie, Junxing Pan, Junqiao Guo, Jianfeng Yin

**Affiliations:** 1 Mental Health Education Center, Anhui Professional College of Art, Hefei, China; 2 Department of Students’ Affair, Huaibei Normal University, Huaibei, China; 3 School of Education, Huaibei Normal University, Huaibei, China; 4 School of Education Science, Qingdao University, Qingdao, China

**Keywords:** art vocational college students, freshmen, psychological health, latent profile analysis, Probabilistic Epigenesis

## Abstract

**Purpose:**

This study aims to investigate the latent categories of psychological health among freshmen in art vocational colleges and examine the influence of demographic variables on these categories, thereby providing an empirical basis for developing targeted intervention strategies.

**Methods:**

A cluster convenience sampling method was employed to recruit 1,685 first-year students from an art vocational college in Anhui Province, China. Participants completed the Chinese College Student Mental Health Screening Scale (CSMHSS). Grounded in the theoretical framework of Probabilistic Epigenesis, latent profile analysis (LPA) was conducted to identify latent psychological health categories. Analysis of variance (ANOVA) and multinomial logistic regression analysis were subsequently performed to examine the associations between demographic variables and latent category membership.

**Results:**

Significant heterogeneity was observed in the psychological health of art vocational college freshmen, with three latent categories identified: the high stressors–low symptoms group (41.8%), the moderate stressors–moderate symptoms group (43.5%), and the low stressors–high symptoms group (14.7%). All 22 dimensions of psychological health were significantly positively correlated with each other (*r* = 0.29–0.86, *p* < 0.01), and each of the 22 dimensions was significantly positively correlated with the total score of psychological health (*r* = 0.62–0.91, *p* < 0.01). ANOVA revealed significant differences across the three latent categories on all 22 dimensions of the CSMHSS (*p* < 0.001). Post-hoc comparisons indicated that the high stressors–low symptoms group scored significantly lower than both the moderate stressors–moderate symptoms group and the low stressors–high symptoms group on all dimensions, while the moderate stressors–moderate symptoms group scored significantly lower than the low stressors–high symptoms group across all dimensions. Multinomial logistic regression analysis demonstrated that gender, place of origin, and academic school significantly influenced the distribution of psychological health subtypes (*p* < 0.05), whereas ethnicity and only-child status did not (*p* > 0.05). Specifically, male students exhibited higher levels of psychological health (OR = 1.790, *p* < 0.01), while freshmen from small towns (OR = 0.528, *p* < 0.05) and those from the School of Fine Arts (OR = 0.429, *p* < 0.05) demonstrated lower levels of psychological health.

**Conclusion:**

This study reveals significant group heterogeneity in the psychological health of art vocational college freshmen. Female students, freshmen from small towns, and those enrolled in the School of Fine Arts warrant particular attention. A tiered and differentiated mental health intervention system should be developed based on latent profile characteristics to address the specific needs of each subgroup.

## Introduction

College freshmen represent a distinct population group in the midst of transitioning from secondary school to university. Due to changes in their surrounding environment, they face not only psychological adaptation but also pressures related to future career exploration, self-concept development, and interpersonal relationship adjustment (Zhang et al., 2024). Recent domestic and international surveys indicate that the psychological health status of college freshmen is concerning (Dong J. et al., 2024). For instance, Zhou et al. found that the one-year prevalence of suicidal ideation among college freshmen in Wuhan, China, was 27.5% (Zhou et al., 2025). Shi administered psychological questionnaires to 11,955 college freshmen enrolled between 2016 and 2019 at a university in Nanjing, China, reporting a detection rate of 22.58% for students potentially exhibiting serious psychological problems (Shi and Ma, 2020). Furthermore, Kiekens et al. surveyed 20,842 first-year students across nine countries and found the past-year prevalence of non-suicidal self-injury to be 8.4%(Kiekens et al., 2023). Consequently, the freshman adaptation period represents a high-incidence phase for psychological problems, warranting significant attention.

Previous research on the psychological health of college freshmen has predominantly focused on general undergraduate populations, with notably insufficient attention given to students in vocational colleges, particularly those in the arts (Willoughby et al., 2020). Freshmen in art vocational colleges constitute a specific subgroup within the broader college freshman population. Their psychological health status exhibits both common issues shared by all freshmen and significant group heterogeneity attributable to their unique disciplinary attributes, training models, and individual characteristics (Feng and Wang, 2024). From a psychological trait perspective, this group is characterized by high sensitivity, manifesting as heightened awareness of environmental stimuli and interpersonal interactions. While this trait can facilitate artistic creation, it also predisposes individuals to negative emotions such as loneliness, anxiety, and low self-esteem (Jadhav, 2023). Research indicates that the detection rate of depressive symptoms among art majors is higher than that among non-art majors (Vaag et al., 2021). Furthermore, art students in vocational colleges demonstrate more pronounced psychological distress due to the dual pressures of their educational level and relatively limited career prospects (Chen,2023). Wang (2023) surveyed 670 art vocational college students and found that their mean loneliness score exceeded the normative level. Zhu (2018) reported that 15% of art vocational college students experienced psychological problems stemming from employment pressure, 10% exhibited mild psychological issues, and 5% required professional psychological intervention and treatment. Given these characteristics, the psychological health issues of art vocational college freshmen warrant urgent attention. To conduct scientifically sound research in this area, the primary tasks are to ensure the appropriateness of measurement instruments and the validity of research methodologies.

However, existing research on the psychological health of college freshmen has certain limitations with respect to the measurement instruments employed. Previous studies have commonly used symptom checklists (e.g., SCL-90), the University Personality Inventory (UPI), and the Cattell Sixteen Personality Factor Questionnaire (16PF)—scales originally developed abroad. Although these tools have undergone Chinese revisions, limitations remain in their applicability within the Chinese cultural context, potentially leading to measurement errors and biased interpretation of results (Huang and Li, 2009; Shan, 1998; Long et al., 2024). Moreover, such scales primarily focus on screening for symptoms of individual psychological problems, providing insufficient assessment of broader developmental and adaptive issues. Consequently, they struggle to comprehensively reflect the overall state of freshmen’s psychological health. In contrast, the Chinese College Student Mental Health Screening Scale is a mature instrument developed specifically for the characteristics of Chinese university students. It retains the core function of screening for psychological problem symptoms while enhancing coverage of key issues pertinent to Chinese students, such as academic stress, interpersonal difficulties, and internet addiction. This makes it more suitable for assessing the transitional adaptation needs of freshmen moving from high school to university (Yi et al., 2022).

In addition to the choice of measurement instruments, research methodology also influences the identification of heterogeneous characteristics in freshmen’s psychological health. Regarding research methodology, existing studies have primarily employed variable-centered approaches, using cut-off scores on scales to classify individuals as psychologically healthy or unhealthy (Nadire et al., 2023). However, this method cannot distinguish between individuals who obtain the same total score but exhibit different response patterns on specific items, making it difficult to effectively capture the heterogeneous characteristics of psychological health within the freshman population. This limitation hinders in-depth identification and analysis of the psychological features of different subgroups. Latent profile analysis (LPA) is a statistical method for classifying individuals and identifying group heterogeneity based on their different response pat terns on continuous variables. This method can identify subgroups that share similar relationships/horizontal patterns in the variable system, allowing for further research on subgroups of various characteristics or the adoption of different intervention approaches (Su et al., 2015; Wen et al., 2023). Adopting this method requires corresponding theoretical support to justify the existence of latent categories of psychological health.

Probabilistic Epigenesis provides a solid theoretical foundation for conducting latent profile analysis of psychological health among freshmen in art vocational colleges. This theory posits that the complexity of an individual’s growth environment contributes to divergences in developmental trajectories. Over time, however, individuals tend to develop in specific directions, gradually coalescing into stable categories (Gottlieb, 2007). Individuals within the same category exhibit greater internal homogeneity, whereas the boundaries between different categories become increasingly distinct. This theoretical perspective challenges the traditional conceptualization of psychological health as a continuous variable, substantiates the existence of latent psychological health categories, and provides a theoretical rationale for employing individual-centered approaches such as latent profile analysis (LPA).

The theoretical framework of Probabilistic Epigenesis has received empirical validation in research on university students’ psychological health. Liu Runxiang assessed the psychological health status of 33,250 undergraduate students at a university in Jiangxi Province, China, and identified five latent categories: a severe psychological problem group, a general psychological problem group, a low self-harm/high internet addiction group, an academic-employment stress group, and a psychologically healthy group (Liu et al., 2024). This finding confirms the presence of categorical differentiation in university students’ psychological health. Dong Junqiang investigated the psychological health status of 2,552 freshmen at a university in Zhejiang Province, China, and identified significant heterogeneity, classifying participants into three categories: a psychologically healthy group, a psychological distress group, and a psychological risk group (Dong J.Q. et al., 2024). These studies suggest that college students’ psychological health is not uniformly distributed but follows a “healthy–transitional–distressed” differentiation pattern: namely, a state of sound psychological health, a borderline intermediate state at risk, and a state of marked psychological distress, featuring intra-class homogeneity and significant inter-class heterogeneity.

Based on the core tenets of Probabilistic Epigenesis and specific to freshmen in art vocational colleges, this group exhibits heightened sensitivity to evaluations of their creative work and interpersonal perceptions (Meng and Ayob, 2025). This dispositional tendency, in conjunction with individual differences in emotion regulation capacity, naturally engenders distinct psychological health states (Yu and Shi, 2025). Some students are able to channel their sensitive psychological traits into creative advantages (Licitra, 2015), effectively managing stress and corresponding to the “healthy” state. Others, possessing moderate regulatory capacity, remain susceptible to environmental adaptation and academic pressures without experiencing persistent distress, corresponding to the “transition” state. A further subset of students, characterized by deficient regulatory capacity, become entrenched in negative emotional states, corresponding to the “distress” category. Moreover, certain art disciplines (e.g., sculpture, painting) emphasize independent creative work, wherein prolonged isolation may precipitate insufficient social interaction. When compounded by the dual pressures of vocational college credential-related identity crises and constrained career prospects the coping trajectories of students become further differentiated (Verma and Jha, 2025; Yali and Nasri, 2025).

In summary, grounded in the theoretical framework of Probabilistic Epigenesis, the present study employs the indigenously developed Chinese College Student Mental Health Screening Scale (CSMHSS) and adopts a individual-centered methodological approach—latent profile analysis (LPA)—to investigate the latent categories of psychological health among freshmen in art vocational colleges and the predictive effects of demographic variables on these categories. This investigation aims to inform targeted guidance and intervention strategies tailored to the distinct psychological health profiles of art vocational college freshmen, while simultaneously addressing the current gap in the research literature concerning the psychological health of this specific population.

## Methods

### Participants

A cluster convenience sampling method was employed to recruit first-year students from an art vocational college in Anhui Province, China, between September and October 2025. Group testing was conducted at the class level, with homeroom teachers who had received training from the university’s Mental Health Education Center serving as test administrators. A total of 1,685 questionnaires were distributed. After excluding invalid responses (e.g., incomplete submissions due to premature withdrawal or patterned responding), 1,677 valid questionnaires were retained, yielding an effective response rate of 99.53%. The mean age of the participating art vocational college freshmen was (18.46 ± 1.67) years. The sample comprised 619 male students (36.9%) and 1,058 female students (63.1%); 417 only-child students (24.9%) and 1,260 non-only-child students (75.1%). Regarding place of origin, 806 students (48.1%) were from rural areas, 354 (21.1%) from small towns, 365 (21.8%) from small-to-medium-sized cities, and 152 (9.1%) from large cities. In terms of academic departments, the sample included 448 students from the School of Fine Arts (including majors in Fine Arts Design and Painting), 591 students from the College of Comprehensive Arts (including majors in Performing Arts, Transportation Services, and Film and Media Technology), 187 students from the Dance Academy (including majors in Dance Performance and International Standard Dance), 237 students from the School of Drama and Media (including majors in Film and Television Directing, Broadcasting and Hosting, and Character Image Design), 39 students from the College of Chinese Opera (including the Huangmei Opera Performance major), and 175 students from the School of Music (including majors in Music Performance and Modern Pop Music).

All participating freshmen were informed of the purpose and significance of the survey and provided written informed consent prior to formal assessment. The study procedures complied with national ethical standards for biomedical research involving human subjects.

### Measures

The Chinese College Student Mental Health Screening Scale (CSMHSS), developed by Fang Xiaoyi and colleagues and subsequently revised by Yi, was employed in this study (Fang et al., 2018; Yi et al., 2022). This scale, designed to assess symptoms of mental health problems among Chinese university students, has become a mainstream instrument for mental health screening in Chinese higher education institutions. It comprises three levels of screening. Level 1 screening includes two indicators: severe psychotic symptoms (e.g., hallucinations and delusions) and suicidal behavior and ideation. Level 2 screening assesses common psychological problems, categorized into internalizing and externalizing problems. Level 3 screening addresses developmental disturbances. These three screening levels primarily reflect the sources of students’ psychological distress and indicate potential underlying psychological problems. The scale consists of 96 items, each rated on a 4-point scale (1 = not at all like me, 4 = very much like me). The scale encompasses 22 dimensions: school adaptation difficulties (4 items; sample item: “I cannot adapt to university life”); employment pressure (4 items; sample item: “I am afraid of facing employment issues”); academic pressure (4 items; sample item: “I work very hard but still cannot keep up with my studies”); romantic relationship concerns (4 items; sample item: “My family obstructs my romantic relationship”); interpersonal difficulties (5 items; sample item: “I feel unwelcome among my classmates”); sensitivity (4 items; sample item: “Other people’s words and actions easily hurt my feelings”); eating problems (4 items; sample item: “I engage in vomiting or fasting behaviors”); paranoia (4 items; sample item: “I always blame others for causing trouble”); impulsivity (4 items; sample item: “I often regret things as soon as I do them”); compulsivity (4 items; sample item: “I cannot continue with other things unless I repeatedly think about or do certain things”); self-injurious behavior (4 items; sample item: “I deliberately burn or scald myself”); depression (6 items; sample item: “I feel very low in mood when I wake up in the morning”); social anxiety (5 items; sample item: “I am overly nervous when talking to others”); hostile aggression (5 items; sample item: “I often lose my temper at others for no reason”); somatization (5 items; sample item: “I feel nauseous or have a stomachache”); internet addiction (5 items; sample item: “Excessive internet use has affected my normal study and life”); low self-esteem (5 items; sample item: “I always feel that I am inferior to others”); dependency (4 items; sample item: “I need someone else to make decisions for me in everything”); anxiety (4 items; sample item: “I constantly worry that something bad will happen and feel uneasy inside”); sleep disturbances (4 items; sample item: “I have difficulty falling asleep or wake up early”); hallucinations and delusions (4 items; sample item: “I always feel that someone wants to harm me”); and suicidal intent (4 items; sample item: “I have considered the methods or timing of suicide”). Scores for each dimension are calculated by summing the relevant items, with higher scores indicating more severe symptoms or distress in that particular domain. Cronbach’s *α* coefficients for each dimension of the scale are presented in Table 1. In the present study, the overall Cronbach’s α coefficient of the scale was 0.969. Confirmatory factor analysis (CFA) indicated good structural validity, with the following model fit indices: *χ*^2^(199) = 2,078.941, CFI = 0.918, TLI = 0.905, RMSEA = 0.075, SRMR = 0.045.

**Table 1:** Cronbach’s *α* coefficients for each dimension of the Chinese college student mental health screening scale.

Dimension name	Cronbach’s *α* coefficient
1. School adaptation difficulties	0.65
2. Employment pressure	0.90
3. Academic pressure	0.85
4. Romantic relationship concerns	0.60
5. Interpersonal difficulties	0.83
6. Sensitivity	0.84
7. Eating problems	0.57
8. Paranoia	0.83
9. Impulsivity	0.81
10. Compulsivity	0.78
11. Self-injurious behavior	0.77
12. Depression	0.84
13. Social anxiety	0.83
14. Hostile aggression	0.77
15. Somatization	0.79
16. Internet addiction	0.85
17. Inferiority	0.88
18. Dependency	0.82
19. Anxiety	0.82
20. Sleep disturbances	0.72
21. Hallucinations and delusions	0.76
22. Suicidal intent	0.85

In this study, the reliability coefficients for three dimensions—school adaptation difficulties (*α* = 0.65), romantic relationship concerns (*α* = 0.60), and eating problems (*α* = 0.57)—were relatively low. This is primarily attributable to the pronounced professional heterogeneity among art vocational college freshmen, with significant variations across different majors in terms of training models, physical appearance requirements, and romantic experiences. Additionally, the physical and mental states of freshmen during the adaptation period are not yet fully stable, which may introduce response variability. These factors collectively contribute to the lower internal consistency of the three dimensions. Slightly lower reliability in these dimensions is a normal phenomenon for this specific population (art vocational college freshmen) within a particular assessment context. Moreover, the overall scale reliability (*α* = 0.969) is high, indicating that the overall structure is stable and reliable. Therefore, the lower reliability of these three dimensions does not substantially affect the rigor or credibility of the study’s conclusions.

### Quality control

Standardized testing instructions for administrators and guidelines for participants were developed. All test administrators received uniform training, and on-site guidance was provided to participants. The purpose of the assessment and the anonymity of responses were emphasized to alleviate participants’ concerns, ensuring that data collection procedures were standardized and that the collected data were accurate and reliable.

### Data analysis

First, data cleaning was performed using SPSS25.0, followed by skewness and kurtosis tests to verify whether the data followed a normal distribution. According to Kline, skewness values within ±3 and kurtosis values within ±10 indicate that the sample data are approximately normally distributed (Kline, 2023). Subsequently, descriptive statistics and correlation analysis were conducted on the 22 dimensions of psychological health. Second, latent profile analysis (LPA) was conducted using Mplus7.4. Model fit for LPA was evaluated using the Akaike information criterion (AIC), Bayesian information criterion (BIC), sample-size-adjusted BIC (aBIC), and entropy index, as well as the Lo–Mendell–Rubin likelihood ratio test (LMR) and the bootstrapped likelihood ratio test (BLRT). Lower AIC, BIC, and aBIC values indicate better model fit. Entropy values closer to 1 reflect higher classification accuracy, with entropy ≥ 0.8 indicating classification accuracy exceeding 90%. Significant *p*-values (*p* < 0.05) for both LMR and BLRT suggest that a k-class model provides a significantly better fit than a k-1 class model (Qiu, 2008). Finally, multinomial logistic regression analysis was performed using SPSS 25.0 to examine the predictive effects of various demographic variables on the latent psychological health categories identified through LPA.

Effect sizes for F-tests are reported using partial eta squared (*η*^2^) values, where *η*^2^ = 0.01 indicates a small effect, *η*^2^ = 0.06 indicates a medium effect, and *η*^2^ = 0.14 indicates a large effect (Cohen, 2013). Statistical significance was set at *p* < 0.05.

## Results

### Normality test

Statistical analysis of the distribution characteristics of the sample data revealed that skewness values for the 22 dimensions of the Chinese College Student Mental Health Screening Scale (CSMHSS) ranged from −0.066 to 1.801, all falling within ±3. Kurtosis values ranged from −0.780 to 3.346, all falling within ±10. These findings indicate that the data are approximately normally distributed, satisfying the normality assumptions required for regression analyses and subsequent statistical procedures.

### Common method biases test

Given that data collected through self-report methods may be susceptible to common method bias, stringent procedural controls were implemented. Additionally, Harman’s single-factor test was conducted to assess common method bias. The results extracted three factors with eigenvalues greater than 1, with the first factor accounting for 30.73% of the total variance, below the critical threshold of 40% (Zhou and Long, 2004). This indicates that common method bias is not a serious concern in this study.

### Descriptive statistics and correlation analysis

Descriptive statistics and correlation analyses for all variables are presented in Tables 2 and 3. The correlation results indicated that all pairwise correlations among the 22 dimensions of psychological health—namely, school adaptation difficulties, employment pressure, academic pressure, romantic relationship concerns, interpersonal difficulties, sensitivity, eating problems, paranoia, impulsivity, compulsivity, self-injurious behavior, depression, social anxiety, hostile aggression, somatization, internet addiction, low self-esteem, dependency, anxiety, sleep disturbances, hallucinations and delusions, and suicidal intent—were significantly positive (*r* = 0.29–0.86, *p* < 0.01). Furthermore, each of the 22 dimensions was significantly positively correlated with the total psychological health score (*r* = 0.62–0.91, *p* < 0.01).

**Table 2:** Descriptive statistics and correlation analysis of variables (*n* = 1,677, Part1/2).

Variable	1	2	3	4	5	6	7	8	9	10	11
1. School adaptation difficulties	1										
2. Employment pressure	0.53^**^	1									
3. Academic pressure	0.51^**^	0.76^**^	1								
4. Romantic relationship concerns	0.43^**^	0.49^**^	0.50^**^	1							
5. Interpersonal difficulties	0.62^**^	0.57^**^	0.63^**^	0.56^**^	1						
6. Sensitivity	0.54^**^	0.60^**^	0.61^**^	0.52^**^	0.68^**^	1					
7. Eating problems	0.46^**^	0.40^**^	0.40^**^	0.45^**^	0.54^**^	0.57^**^	1				
8. Paranoia	0.57^**^	0.50^**^	0.52^**^	0.52^**^	0.70^**^	0.75^**^	0.64^**^	1			
9. Impulsivity	0.54^**^	0.57^**^	0.59^**^	0.51^**^	0.63^**^	0.76^**^	0.62^**^	0.74^**^	1		
10. Compulsivity	0.51^**^	0.57^**^	0.57^**^	0.48^**^	0.61^**^	0.78^**^	0.62^**^	0.73^**^	0.75^**^	1	
11. Self-injurious behavior	0.39^**^	0.30^**^	0.29^**^	0.40^**^	0.49^**^	0.49^**^	0.72^**^	0.59^**^	0.52^**^	0.54^**^	1
12. Depression	0.56^**^	0.60^**^	0.60^**^	0.53^**^	0.65^**^	0.78^**^	0.68^**^	0.78^**^	0.77^**^	0.80^**^	0.63^**^
13. Social anxiety	0.53^**^	0.54^**^	0.53^**^	0.43^**^	0.61^**^	0.71^**^	0.57^**^	0.66^**^	0.68^**^	0.73^**^	0.52^**^
14. Hostile aggression	0.52^**^	0.40^**^	0.40^**^	0.43^**^	0.58^**^	0.63^**^	0.69^**^	0.74^**^	0.71^**^	0.65^**^	0.69^**^
15. Somatization	0.40^**^	0.38^**^	0.38^**^	0.39^**^	0.51^**^	0.58^**^	0.73^**^	0.61^**^	0.61^**^	0.65^**^	0.67^**^
16. Internet addiction	0.54^**^	0.52^**^	0.50^**^	0.45^**^	0.52^**^	0.66^**^	0.53^**^	0.65^**^	0.71^**^	0.67^**^	0.46^**^
17. Inferiority	0.52^**^	0.61^**^	0.61^**^	0.53^**^	0.65^**^	0.82^**^	0.60^**^	0.76^**^	0.76^**^	0.80^**^	0.58^**^
18. Dependency	0.50^**^	0.55^**^	0.55^**^	0.48^**^	0.58^**^	0.72^**^	0.57^**^	0.67^**^	0.77^**^	0.73^**^	0.51^**^
19. Anxiety	0.48^**^	0.54^**^	0.55^**^	0.46^**^	0.60^**^	0.78^**^	0.66^**^	0.76^**^	0.77^**^	0.82^**^	0.59^**^
20. Sleep disturbances	0.47^**^	0.47^**^	0.46^**^	0.42^**^	0.50^**^	0.64^**^	0.61^**^	0.63^**^	0.68^**^	0.72^**^	0.51^**^
21. Hallucinations and delusions	0.41^**^	0.34^**^	0.35^**^	0.42^**^	0.53^**^	0.62^**^	0.67^**^	0.66^**^	0.59^**^	0.64^**^	0.63^**^
22. Suicidal intent	0.36^**^	0.29^**^	0.29^**^	0.39^**^	0.48^**^	0.50^**^	0.64^**^	0.54^**^	0.51^**^	0.54^**^	0.70^**^
Total score	0.66^**^	0.68^**^	0.68^**^	0.62^**^	0.76^**^	0.86^**^	0.76^**^	0.85^**^	0.86^**^	0.87^**^	0.69^**^
*M*	7.56	9.24	8.83	6.74	7.00	7.61	6.08	6.99	7.60	7.39	5.44
SD	2.41	3.18	2.83	2.42	2.31	2.77	1.99	2.45	2.67	2.68	1.99

^**^p < 0.01. *M*, Mean; SD, Standard Deviation.

**Table 3:** Descriptive statistics and correlation analysis of variables (*n* = 1,677, Part2/2).

Variable	12	13	14	15	16	17	18	19	20	21	22
12. Depression	1										
13. Social anxiety	0.77^**^	1									
14. Hostile aggression	0.70^**^	0.59^**^	1								
15. Somatization	0.69^**^	0.59^**^	0.67^**^	1							
16. Internet addiction	0.69^**^	0.65^**^	0.63^**^	0.55^**^	1						
17. Inferiority	0.86^**^	0.78^**^	0.66^**^	0.62^**^	0.68^**^	1					
18. Dependency	0.74^**^	0.73^**^	0.63^**^	0.56^**^	0.70^**^	0.78^**^	1				
19. Anxiety	0.82^**^	0.73^**^	0.71^**^	0.74^**^	0.68^**^	0.81^**^	0.74^**^	1			
20. Sleep disturbances	0.70^**^	0.62^**^	0.62^**^	0.70^**^	0.65^**^	0.66^**^	0.61^**^	0.74^**^	1		
21. Hallucinations and delusions	0.64^**^	0.61^**^	0.65^**^	0.66^**^	0.52^**^	0.67^**^	0.59^**^	0.69^**^	0.62^**^	1	
22. Suicidal intent	0.61^**^	0.52^**^	0.63^**^	0.67^**^	0.45^**^	0.57^**^	0.49^**^	0.61^**^	0.54^**^	0.71^**^	1
Total score	0.91^**^	0.83^**^	0.80^**^	0.76^**^	0.79^**^	0.90^**^	0.83^**^	0.89^**^	0.79^**^	0.75^**^	0.68^**^
*M*	9.09	7.04	6.25	6.32	9.53	8.82	6.98	7.07	7.46	5.83	5.56
SD	3.36	2.61	2.28	2.56	3.48	3.35	2.56	2.70	2.83	2.22	2.47

^**^p < 0.01. *M*, Mean; SD, Standard Deviation.

### Latent profile analysis of psychological health among art vocational college freshmen

Based on a one-class model, latent profile analysis was conducted by sequentially specifying models with one to five classes. Fit indices for each model are presented in Table 4. As the number of classes increased, AIC, BIC, and aBIC values progressively decreased. The LMR test was statistically significant for the two-class and three-class models (*p* < 0.05) but not for the four-class and five-class models (*p* > 0.05); consequently, the four-class and five-class solutions were excluded. The BLRT was statistically significant for all five models (*p* < 0.05). Entropy values for the two-class and three-class models were 0.971 and 0.970, respectively, indicating high and comparable classification accuracy. However, given the lower AIC, BIC, and aBIC values for the three-class model, and considering the comprehensive model fit information, the three-class solution was selected as the optimal latent profile model.

**Table 4:** Fit indices for latent profile analysis of psychological health among art vocational college freshmen.

Number of profiles	AIC	BIC	aBIC	LMR (*p*)	BLRT (*p*)	Entropy	Proportions
Class 1	175,590.424	175,829.113	175,689.331	–	–	–	–
Class 2	156,992.135	157,355.594	157,142.744	0.000	0.000	0.971	0.505/0.495
Class 3	150,457.254	150,945.482	150,659.565	0.000	0.000	0.970	0.418/0.435/0.147
Class 4	147,487.979	148,100.977	147,741.991	0.597	0.000	0.947	0.282/0.348/0.271/0.099
Class 5	145,488.197	146,225.964	145,793.911	0.267	0.000	0.955	0.258/0.254/0.329/0.023/0.136

AIC, Akaike information criteria; BIC, Bayesian information criteria; aBIC, adjusted Bayesian information criteria; LMR-LRT, Lo-MendellRubin likelihood ratio Test; BLRT, bootstrap likelihood ratio test.

In the three-class model, the average latent class probabilities for most likely class membership were 98.8% for Class 1, 98.5% for Class 2, and 98.5% for Class 3, indicating high reliability of the latent profile analysis results (see Table 7).

### Classification and naming of latent profiles

**Figure 1:**
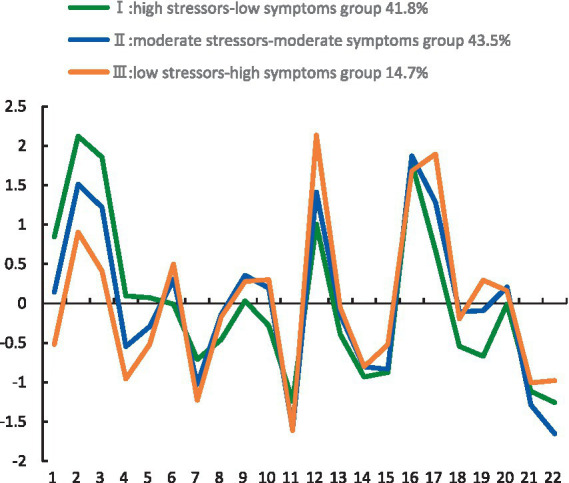
Standardized scores of potential categories for psychological health among freshmen in Chinese art vocational colleges across various dimensions. The horizontal axis represents the dimensions for psychological health among freshmen in Chinese art vocational colleges, and the vertical axis represents the standardized scores of the samples in each dimension. 1 = school adaptation difficulties; 2 = employment pressure; 3 = academic pressure; 4 = romantic relationship concerns; 5 = interpersonal difficulties; 6 = sensitivity; 7 = eating problems; 8 = paranoia; 9 = impulsivity; 10 = compulsivity; 11 = self-injurious behavior; 12 = depression; 13 = social anxiety; 14 = hostile aggression; 15 = somatization; 16 = internet addiction; 17 = low self-esteem; 18 = dependency; 19 = anxiety; 20 = sleep disturbances; 21 = hallucinations and delusions; 22 = suicidal intent.

As shown in Figure 1, the 22 dimensions of psychological health included in this study comprise two distinct types of indicators. Dimensions such as depression, anxiety, self-injurious behavior, and hallucinations/delusions reflect the level of clinical psychological symptoms and serve as criteria for judging the severity of psychological health impairment. In contrast, dimensions such as employment stress, academic stress, school adjustment difficulties, romantic relationship distress, and interpersonal distress reflect situational, externally triggered stress and adaptation challenges. These are important precipitating factors for symptoms but are not equivalent to clinical symptoms themselves. Using clinical symptoms and stressors as the basis for nomenclature, and based on the differences in standard deviations from the mean (as illustrated in Figure 1) for each psychological health subgroup across the 22 dimensions, we designated the three subgroups as the “high stressors–low symptoms group,” the “moderate stressors–moderate symptoms group,” and the “the low stressors–high symptoms group.”

The high stressors–low symptoms group (41.8% of the total sample, *n* = 701) scored significantly higher than the other two groups on external real-world stress dimensions such as school adjustment difficulties, employment stress, and academic stress, while scoring below the sample average on symptom dimensions including depression, anxiety, self-injurious behavior, suicidal ideation, and somatization. This suggests that this subgroup does not exhibit severe psychological or behavioral symptoms and generally maintains good psychological health. The moderate stressors–moderate symptoms group (43.5% of the total sample, *n* = 729) scored at moderate levels across all psychological health dimensions, demonstrating both a certain degree of perceived external stress and mild-to-moderate psychological symptoms. This subgroup represents a “transitional state” of psychological health, with symptoms below the pathological threshold. The low stressors–high symptoms group (14.7% of the total sample, *n* = 247) scored significantly higher than the other two groups on symptom dimensions such as depression, anxiety, self-injurious behavior, suicidal ideation, somatization, and hallucinations/delusions, with scores on some dimensions far exceeding the sample average, indicating clear psychopathological manifestations. However, this group scored significantly lower on external stress dimensions (school adjustment difficulties, employment stress, academic stress), suggesting that their psychological problems are no longer driven primarily by external stress but instead are characterized by predominant psychopathological symptoms.

### Characteristics and differences across psychological health subtypes on each dimension

Building upon the identification of three psychological health subtypes among art vocational college freshmen, analysis of variance (ANOVA) was conducted to examine the characteristics and differences across the three subtypes on each of the 22 psychological health dimensions, thereby validating the results of the latent profile analysis. As shown in Table 5, significant differences were observed across the three psychological health subtypes on all 22 dimensions (*p* < 0.001). More importantly, post-hoc comparisons revealed that the high stressors–low symptoms group scored significantly lower than both the moderate stressors–moderate symptoms group and the low stressors–high symptoms group on all 22 dimensions. Furthermore, the moderate stressors–moderate symptoms group scored significantly lower than the low stressors–high symptoms group on all 22 dimensions. These findings provide robust validation for the three-class model derived from the latent profile analysis of psychological health among art vocational college freshmen.

**Table 5:** Comparison of differences in freshmen in art vocational colleges across latent profiles of psychological health.

Variable	The high stressors–low symptoms group (I, *n* = 701)	The moderate stressors–moderate symptoms group (II, *n* = 729)	The low stressors–high symptoms group (III, *n* = 247)	*F* statistic	*η* ^2^	Post-Hoc comparisons
1	6.05 ± 2.077	8.27 ± 1.815	9.73 ± 2.200	396.225^***^	0.321	I < II < III
2	7.19 ± 2.893	10.18 ± 2.364	12.27 ± 2.169	443.552^***^	0.346	I < II < III
3	6.95 ± 2.536	9.77 ± 2.014	11.40 ± 2.244	458.605^***^	0.354	I < II < III
4	5.39 ± 1.724	7.30 ± 2.083	8.94 ± 2.758	319.101^***^	0.276	I < II < III
5	5.37 ± 1.652	7.65 ± 1.633	9.74 ± 2.076	685.117^***^	0.450	I < II < III
6	5.30 ± 1.477	8.49 ± 1.711	11.56 ± 2.027	1,467.884^***^	0.637	I < II < III
7	4.67 ± 0.973	6.63 ± 1.553	8.45 ± 2.230	689.505^***^	0.452	I < II < III
8	4.90 ± 1.247	7.87 ± 1.470	10.36 ± 1.985	1,486.366^***^	0.640	I < II < III
9	5.34 ± 1.530	8.55 ± 1.638	11.17 ± 1.949	1,365.480^***^	0.620	I < II < III
10	5.05 ± 1.337	8.35 ± 1.563	11.20 ± 1.888	1,736.568^***^	0.675	I < II < III
11	4.21 ± 0.554	5.83 ± 1.761	7.78 ± 2.584	514.243^***^	0.381	I < II < III
12	6.19 ± 1.417	10.05 ± 1.816	14.47 ± 2.354	2,222.347^***^	0.726	I < II < III
13	4.96 ± 1.346	7.86 ± 1.752	10.57 ± 2.287	1,152.218^***^	0.579	I < II < III
14	4.48 ± 0.904	6.93 ± 1.675	9.23 ± 2.325	1,003.996^***^	0.545	I < II < III
15	4.53 ± 1.156	6.90 ± 2.035	9.73 ± 2.620	797.200^***^	0.488	I < II < III
16	6.88 ± 2.402	10.69 ± 2.386	13.69 ± 2.833	839.130^***^	0.501	I < II < III
17	5.90 ± 1.283	9.86 ± 1.914	14.04 ± 2.410	2,156.253^***^	0.720	I < II < III
18	4.83 ± 1.218	7.91 ± 1.711	10.33 ± 2.164	1,286.902^***^	0.606	I < II < III
19	4.70 ± 1.060	7.94 ± 1.575	11.20 ± 1.952	2,062.925^***^	0.711	I < II < III
20	5.30 ± 1.727	8.37 ± 2.083	10.94 ± 2.286	881.491^***^	0.513	I < II < III
21	4.31 ± 0.800	6.27 ± 1.794	8.83 ± 2.454	780.291^***^	0.482	I < II < III
22	4.19 ± 0.760	5.75 ± 2.056	8.90 ± 3.269	561.571^***^	0.402	I < II < III

^***^p < 0.001. 1, school adaptation difficulties; 2, employment pressure; 3, academic pressure; 4, romantic relationship concerns; 5, interpersonal difficulties; 6, sensitivity; 7, eating problems; 8, paranoia; 9, impulsivity; 10, compulsivity; 11, self-injurious behavior; 12, depression; 13, social anxiety; 14, hostile aggression; 15, somatization; 16, internet addiction; 17, low self-esteem;18, dependency; 19, anxiety;20, sleep disturbances; 21, hallucinations and delusions; 22, suicidal intent. I, the high stressors–low symptoms group; II, the moderate stressors–moderate symptoms group; III, the low stressors–high symptoms group.

### Influence of demographic variables on psychological health categories among art vocational college freshmen

To examine the predictive effects of gender, ethnicity, place of origin, only-child status, and academic school on psychological health subtypes among art vocational college freshmen, multinomial logistic regression analysis was conducted. The latent profile analysis classifications served as the dependent variable, with the low stressors–high symptoms group designated as the reference category. Independent variables included gender (female as reference), ethnicity (ethnic minority as reference), place of origin (large city as reference), only-child status (only child as reference), and department (music school as reference). The results (see Table 6) revealed that gender, place of origin, and academic school significantly influenced the distribution of psychological health subtypes among art vocational college freshmen (*p* < 0.05), whereas ethnicity and only-child status did not (*p* > 0.05). Specifically, male students were more likely to be classified into the high stressors–low symptoms group compared to female students. Freshmen from small towns were more likely to be classified intothe low stressors–high symptoms group compared to those from large cities. Additionally, freshmen from the School of Fine Arts were more likely to be classified into the low stressors–high symptoms group compared to those from the School of Music.

**Table 6:** Multinomial logistic regression analysis of demographic variables on three latent psychological health categories among art vocational college freshmen (*n* = 1,677).

Variables	Demographic variables	The high stressors–low symptoms group			The moderate stressors–moderate symptoms group		
		*β*	OR	95%CI	*β*	OR	95%CI
Gender	Male	0.582	1.790^**^	1.288, 2.488	0.321	1.378	0.994, 1.912
	Female		–			–	
Ethnicity	Han Chinese	0.023	1.024	0.362, 2.891	0.832	2.299	0.711, 7.435
	Ethnic minority		–			–	
Place of origin	Rural areas	−0.302	0.739	0.407, 1.344	0.071	1.074	0.589, 1.956
	Small town	−0.639	0.528^*^	0.284, 0.981	−0.415	0.661	0.355, 1.231
	Medium-sized city	−0.402	0.669	0.359, 1.247	−0.246	0.782	0.418, 1.463
	Big city		–			–	
Only-child status	Yes		–			–	
	No	−0.151	0.860	0.591, 1.253	−0.270	0.763	0.527, 1.105
Department	School of fine arts	−0.846	0.429^*^	0.245, 0.753	−0.411	0.663	0.383, 1.149
	College of comprehensive arts	0.345	1.412	0.805, 2.476	−0.092	0.913	0.519, 1.604
	Dance academy	−0.435	0.647	0.335, 1.250	−0.092	0.912	0.482, 1.727
	School of drama and media	−0.261	0.770	0.417, 1.422	−0.533	0.587	0.317, 1.087
	College of Chinese opera	−0.421	0.656	0.202, 2.131	0.003	1.003	0.327, 3.080
	Music school		–			–	

^*^p < 0.05, ^**^p < 0.01. OR refers to the odds ratio coefficient, and CI (confidence interval) refers to the confidence interval of the OR. The reference categories were as follows: gender (female as reference), ethnicity (ethnic minority as reference), place of origin (large city as reference), only-child status (only child as reference), and department (music school as reference).

**Table 7:** Average latent class probabilities for most likely latent class membership by latent profile.

Number of profiles	Class 1 (%)	Class 2 (%)	Class 3 (%)
Class 1	0.988	0.012	0.000
Class 2	0.009	0.985	0.006
Class 3	0.000	0.015	0.985

Class 1, the high stressors–low symptoms group; Class 2, the moderate stressors–moderate symptoms group; Class 3, the low stressors–high symptoms group.

## Discussion

### Characteristics of psychological health among art vocational college freshmen

Latent profile analysis (LPA) revealed that the psychological health of first-year art-oriented vocational college students comprised three latent classes: the high stressors–low symptoms group (*n* = 701, 41.8%), the moderate stressors–moderate symptoms group (*n* = 729, 43.5%), and the low stressors–high symptoms group (*n* = 247, 14.7%). In the three-class solution, the average posterior probabilities of correct classification were 98.8, 98.5, and 98.5%, respectively, indicating stable features of high intra-class homogeneity and strong inter-class heterogeneity. These findings are consistent with the theoretical predictions of Probabilistic Epigenesis, thereby supporting the credibility of the LPA results in this study.

The Probabilistic Epigenesis perspective posits that individual development diverges due to initial conditions such as genetic endowment, environment, and demographic characteristics. During continuous environmental adaptation, individuals’ psychological traits, coping styles, and external stressors interact dynamically, gradually consolidating developmental trajectories into stable subgroups characterized by high intra-class homogeneity and clear inter-class boundaries. Building on initial differences in gender, hometown origin, and academic major among first-year art-oriented vocational students (Wen et al., 2025; Rahman et al., 2025; Dong et al., 2023), and through the dynamic adjustment of the college adaptation phase, their psychological health does not follow a continuous distribution. Instead, it differentiates into three classes with stable characteristics and clear boundaries (Li et al., 2026; Chen, 2023; Masten and Cicchetti, 2016). Analysis of variance (ANOVA) showed significant differences among the three classes across all 22 dimensions (*p* < 0.001), confirming the explanatory power of the Probabilistic Epigenesis perspective regarding the formation of heterogeneity in college students’ psychological health and providing a solid theoretical foundation for the LPA in this study.

According to the proportions of the three latent classes, 85.3% of freshmen in art vocational colleges were in the “high stressors–low symptoms group” or “the moderate stressors–moderate symptoms group” states, indicating that their psychological health was generally manageable. The remaining 14.7% belonged to the low stressors–high symptoms group, exhibiting multidimensional psychological problems. This finding is largely consistent with previous studies and reflects the general state of psychological health among college freshmen (Liu and Li, 2024; Bruffaerts et al., 2018). Moreover, the three-class solution conforms to the “healthy–transitional–distressed” differentiation pattern of psychological health. That is, psychological health is not a single continuum; rather, during the college adaptation process, initial differences and the interaction between psychological traits and the environment gradually give rise to a healthy state, a transitional intermediate state, and a distressed state. In this pattern, “healthy” denotes minimal psychological symptoms and good overall adaptation; “transitional” denotes a borderline state characterized by moderate stress and mild symptoms, carrying the potential for bidirectional change; and “distressed” denotes a high-risk state featuring prominent multidimensional symptoms and low levels of psychological health, requiring targeted intervention. These three states share high internal homogeneity and clear external differentiation, collectively forming the class structure of college students’ psychological health.

Focusing specifically on freshmen in art vocational colleges: The high stressors–low symptoms group is primarily characterized by developmental problems, with emphasis needed on academic and employment stress. Art-related majors require not only a solid theoretical foundation but also continuous skills training. Students must invest substantial time and effort in practice, which can readily lead to burnout and academic stress (Wang et al., 2024). Given that the labor market demand for art-oriented positions is relatively limited, supply–demand imbalance is common. Furthermore, with the recent rise of emerging fields such as digital art and AI design, some traditional art roles have been displaced. Many art-oriented vocational students lack career planning and face unemployment upon graduation, contributing to high employment stress (Zhao, 2023).

The moderate stressors–moderate symptoms group, the largest proportion of the sample (43.5%), scored at moderate levels across all psychological health dimensions. These students perceive both academic and employment stress while also experiencing mild depression, anxiety, and other psychological symptoms. On the one hand, they can maintain basic academic and daily functioning without reaching clinical pathological levels. On the other hand, because art-related majors emphasize skill proficiency, these students are more prone to accumulating negative emotions due to creative setbacks. Without timely guidance and support, this can gradually escalate into high-risk problems such as depression, low self-esteem, and self-injurious tendencies (Vaag et al., 2021; Yu and Shi, 2025). Therefore, this subgroup represents a prototypical “transitional state” of psychological health and serves as a critical juncture for preventing symptom deterioration and blocking risk escalation (Kaplow, 2021).

The low stressors–high symptoms group exhibited pronounced depressive tendencies and low self-esteem, consistent with multiple previous studies (Wang, 2023; Li and Yang, 2021). First, art students generally show high emotional sensitivity (Song et al., 2025; Farstad and Arnulf, 2024; Furnham and Crump, 2013) and often pursue perfection in their creative work, making them more vulnerable to psychological discrepancy triggered by external negative feedback or unsatisfactory outcomes (Bridges and Schendan, 2019). In the absence of effective emotion regulation mechanisms, the cumulative effect may exacerbate depressive tendencies (Gross, 2002). Second, the nature of art training requires substantial time spent in solitary practice, which objectively reduces opportunities for social interaction (Kaya and Mathieu, 2025). Additionally, some students may experience interpersonal adjustment difficulties in group settings, leading to emotional distress due to social isolation or interpersonal conflicts (Kaplow, 2021). Third, this group faces significant challenges to self-esteem. Societal stereotypes that have long portrayed art students as having poor academic performance in cultural subjects expose them to persistent biases such as “insufficient learning ability” or “opportunists taking shortcuts in college admissions” (Elpus, 2014). This identity crisis not only undermines professional self-confidence but may also, through continuous negative self-suggestion, reduce overall self-worth and ultimately foster low self-esteem.

### Influence of demographic variables on psychological health among art vocational college freshmen

Multinomial logistic regression analysis revealed that gender, place of origin, and academic school significantly influenced the distribution of psychological health subtypes among art vocational college freshmen, whereas ethnicity and only-child status did not.

Compared to female students, male students were more likely to be classified into the low stressors-low symptoms group, indicating higher levels of psychological health among males. This finding diverges from the results reported by Dong et al. In the field of art education, females confront more complex evaluative standards. On one hand, their creative work is expected to embody traditional feminine qualities such as softness and delicacy; on the other hand, it must satisfy professional requirements for artistic innovation. Consequently, female art students experience greater pressure (Ginis et al., 2023). Particularly in performing arts disciplines such as drama and dance, the proliferation of social media has intensified scrutiny of female students’ physical appearance (Azazz, 2025), rendering them more susceptible to body image concerns, eating disorders, and other psychological health issues (Pedalino and Camerini, 2022; Liang et al., 2020). Additionally, due to dual pressures arising from societal gender expectations, females are required not only to maintain academic excellence but also to conform to traditional gender role norms. These multiple social expectations create sustained psychological burden, continuously depleting psychological resources and adversely affecting psychological health (Zheng et al., 2022; Matud, 2004; Luo et al., 2023).

Compared to art vocational college freshmen from large cities, those from small towns were more likely to be classified into the high stressors–high symptoms group, indicating relatively lower levels of psychological health. Students from large cities typically receive quality foundational art education from an early age, whereas those from small towns predominantly undergo traditional examination-oriented training. Upon entering university evaluation systems that emphasize innovative capacity, the latter group is more prone to deficits in professional confidence and adaptation anxiety, culminating in low self-esteem and ability-related apprehension (Meng et al., 2024; Yasmin et al., 2023). Furthermore, families in small towns are more likely to perceive art education as a high-risk investment, with artistic training consuming a substantial portion of household expenditures, thereby subjecting students to pressure for success (Meng et al., 2024; An and Guan, 2024). In contrast, the relatively favorable economic conditions of families in large cities provide students with stronger familial support systems, reducing the pressure for immediate economic returns and alleviating psychological burden.

Compared to students from schools of music, those from schools of fine arts were more likely to be classified into the high stressors–high symptoms group, indicating relatively lower levels of psychological health. Research has demonstrated that music significantly reduces physiological stress indicators such as heart rate and blood pressure, evokes positive emotions, alleviates anxiety and depression (Cao and Zhang, 2023; Mir et al., 2021; Witte et al., 2020; Zou and Phokha, 2025), and facilitates stress release, thereby improving emotional states. In contrast, fine arts creation necessitates sustained exposure of one’s inner world, such as expressing traumatic memories through visual imagery. This self-examining mode of work predisposes individuals to emotional exhaustion. From a neuroscientific perspective, musical activities primarily activate the brain’s reward pathways, promoting dopamine release. Conversely, fine arts creation requires sustained activation of the default mode network (DMN), a neural activity pattern associated with depressive tendencies (Chou et al., 2023). Additionally, fine arts disciplines such as oil painting and sculpture often demand continuously high-intensity work, and irregular routines can readily disrupt circadian rhythms, thereby precipitating emotional disorders (Roles and Kim, 2022).

### Theoretical contributions

The theoretical contributions of this study are threefold. First, it validates the explanatory power of the Probabilistic Epigenesis perspective regarding the formation of heterogeneity in psychological health among freshmen in art vocational colleges, thereby extending the application of this theory to the contexts of vocational education and art-major students. Second, it reveals a three-class latent pattern of “healthy–transitional–distressed,” refining the structural theory of psychological health for freshmen in art vocational colleges. Third, it identifies the predictive effects of gender, hometown origin, and academic major on class differentiation, deepening our understanding of the mechanisms underlying heterogeneity in psychological health.

## Limitations and future directions

Several limitations of this study warrant acknowledgment. First, the research sample was drawn only from freshmen at a single art-oriented vocational college in Anhui Province, which limits the generalizability and applicability of the findings to the broader population of art vocational college students nationwide. Future research should adopt diversified sampling strategies, including multiple art vocational colleges across different provinces, to expand sample coverage and further improve the external validity and generalizability of the conclusions.

Second, this study employed a cross-sectional design, which only captures the latent class characteristics of psychological health among freshmen in art vocational colleges at a single time point and cannot depict the dynamic formation and transition trajectories of these classes. Subsequent studies could conduct longitudinal follow-up investigations to analyze the developmental features of latent classes at different stages of the college years and to clarify the patterns of transition between classes.

Third, this study used only the Chinese College Students Mental Health Screening Scale as a single self-report measure. Although this scale has good local adaptation, self-report methods are susceptible to biases such as social desirability and self-perception inaccuracies. Art students, who may be particularly sensitive to psychological assessment, might exhibit certain response biases. Future research should incorporate multi-dimensional assessment approaches, including clinical interviews and behavioral observations, to achieve a comprehensive and holistic evaluation of psychological health status.

Fourth, this study included only demographic variables such as gender, hometown origin, and academic major, without examining key factors closely related to art vocational college students, such as parenting styles, professional identity, social support, and emotion regulation strategies. Future research should integrate these variables to gain deeper insight into the underlying mechanisms driving the differentiation of latent classes in psychological health.

## Conclusion

Grounded in the theoretical framework of Probabilistic Epigenesis, this study employed latent profile analysis to reveal the heterogeneous characteristics of psychological health among freshmen in art vocational colleges, thereby offering a novel perspective for understanding the psychological health status of this specific population. The empirical findings indicate that the psychological health of art vocational college freshmen can be classified into three latent categories: the high stressors–low symptoms group (41.8%), the moderate stressors–moderate symptoms group (43.5%), and the low stressors–high symptoms group (14.7%). Gender, place of origin, and academic school significantly influenced the distribution of psychological health subtypes, manifesting as follows: male students demonstrated higher levels of psychological health than female students; freshmen from small towns were more susceptible to psychological distress; and students from schools of fine arts exhibited greater psychological health risk compared to those from schools of music. In contrast, ethnicity and only-child status did not affect the distribution of psychological health subtypes among art vocational college freshmen.

### Educational implications

Freshmen in art vocational colleges are situated in a critical transitional period from high school to university. The unique pressures associated with specialized artistic training, compounded by the adaptive challenges of environmental transition, render their psychological health concerns distinct from those of general undergraduate students and non-art majors. Without timely intervention, potential psychological distress may intensify, adversely affecting academic development, career planning, and overall wellbeing. Accordingly, based on the findings of this study and considering the practical characteristics of art vocational education, the following targeted educational recommendations are proposed:

For freshmen in the high stressors–low symptoms group, efforts should focus on maintaining their positive psychological state while providing targeted guidance for academic and employment pressures. First, career planning guidance tailored to the characteristics of art disciplines should be implemented to help students clarify their developmental trajectories and alleviate career-related anxiety. Second, platforms for artistic creation and exchange should be established—through collective creative activities and art clubs—to further strengthen social connections and consolidate psychological health.

For freshmen in the moderate stressors–moderate symptoms group, routine psychological monitoring and targeted intervention should be implemented, with particular emphasis on addressing prominent issues such as internet addiction and interpersonal difficulties. Counselors and mental health education staff should conduct personalized consultations to assess the severity and underlying causes of psychological distress. Simultaneously, peer support should be mobilized to encourage participation in offline social and practical activities, fostering healthy internet use habits and facilitating successful psychological transition from high school to university, thereby preventing further exacerbation of difficulties.

For freshmen in the low stressors–high symptoms group, immediate targeted support and professional intervention mechanisms should be initiated. Teachers from the university’s Mental Health Education Center should conduct one-on-one professional interviews and psychological counseling, focusing on alleviating negative emotions such as depression and low self-esteem. For students presenting more pronounced symptoms, timely referral to professional psychological diagnosis and treatment institutions should be arranged for comprehensive assessment and systematic intervention. Concurrently, collaboration with parents and specialized course instructors should be established to create an inclusive and supportive learning environment. Through early intervention and sustained care, psychological symptoms can be alleviated, assisting students in overcoming psychological difficulties.

In summary, these differentiated intervention strategies are intended to help art vocational college freshmen better adapt to university life, thereby maintaining and enhancing their psychological health.

## Data Availability

The raw data supporting the conclusions of this article will be made available by the authors, without undue reservation.
